# Engineering Small-Molecule Proton-Transfer Ferroelectrics by Crystal Structure Prediction: Design Limits at the Salt–Cocrystal Boundary

**DOI:** 10.1021/acs.chemmater.6c01164

**Published:** 2026-07-12

**Authors:** S. Seyedraoufi, Owen D. W. Hewitt, Simon J. Coles, Graeme M. Day, Kristian Berland

**Affiliations:** † Department of Mechanical Engineering and Technology Management, 56625 Norwegian University of Life Sciences, Ås 1432, Norway; ‡ School of Chemistry and Chemical Engineering, 7423 University of Southampton, Southampton SO17 1BJ, United Kingdom; § Department of Mechanical Engineering and Technology Management, 56625 Norwegian University of Life Sciences, Ås 1432, Norway

## Abstract

Organic molecular ferroelectrics hold significant potential in organic electronics due to their chemical tunability and straightforward fabrication methods. Among these, acid–base proton-transfer (PT) salts are notable for their low coercive fields and fast switching capabilities but are limited by relatively low spontaneous polarization. Using smaller molecular species can in principle increase the polarization, but requires both stabilization of the monovalent salt state and crystal packing that supports ferroelectric PT pathways. Using a crystal-structure prediction (CSP)-based design combined with density functional theory (DFT), we investigate 30 combinations of molecular acids and bases aimed at enhancing the dipole density. We identified several crystal structures with PT-capable hydrogen-bonding networks, and in our initial DFT ranking, three candidate ferroelectric packings and one antiferroelectric. Subsequent experimental work on two representative systems, while confirming the ability of CSP to predict PT-capable packing motifs, found neutral cocrystals rather than the desired monovalent salts supporting ferroelectricity. More detailed computational analysis traced the disparity to the relative stability of protonation states, which is strongly sensitive to the exchange–correlation functional and to vibrational zero-point energy contributions. Thus, while our CSP study correctly identified proton-transfer crystal packing motifs, it failed at the level of protonation-state stability, which we found to be strongly influenced by exchange–correlation choice and vibrational free energy.

## Graphical abstract

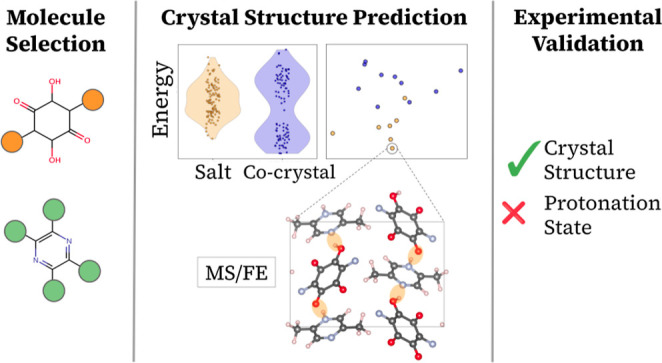

## Introduction

Traditional ceramic and polymer ferroelectrics are widely used in capacitors, sensors, actuators, and memory devices,^1–3^ owing to their piezoelectric and pyroelectric properties and switchable spontaneous polarization (***P***_s_).^4^ There is, however, growing interest in small-molecule ferroelectric and corresponding antiferroelectric materials,^5–7^ which offer low weight, chemical tunability, and compatibility with low-cost and flexible device fabrication.^7–12^ These systems can exhibit fast switching and low-voltage operation,^13,14^ in contrast to polymer ferroelectrics that often suffer from high coercive fields.^10,15^ Their molecular nature also enables extensive design flexibility through functionalization and cocrystal engineering strategies.^16–20^

Proton-transfer (PT) systems are one class of small-molecule ferroelectric materials. Their polarization direction can be reversed through concerted or cascading proton transfer between molecules. This behavior is realized in tautomeric crystals,^9,10,14,21,22^ amphoteric compounds,^23^ and organic acid–base salts.^24–28^ Compared to tautomeric crystals,^10^ acid–base salts tend to have lower coercive fields, which is attractive for low-energy switching and sensing.^5,13^

Designing acid–base PT salts with ferroelectric or antiferroelectric properties constitutes a cocrystal engineering challenge. Changing the acid or base species—through size, shape, acidity, or functionalization—can tune properties such as ***P***_s_, but also modifies intermolecular interactions and crystal packing, potentially disrupting the hydrogen-bonding motifs required for PT switching.^29^ Moreover, chemical modification can alter the preferred protonation state.^30^

For crystal structures that support connected proton-transfer paths, several scenarios can arise (Figure 1). Systems that form neutral cocrystals (NCs) or divalent salts (DSs), rather than monovalent salts (MSs), are typically paraelectric, although an external field can induce a polar MS configuration. When one-dimensional chains of directional hydrogen bonds are present, the resulting structures can adopt either polar ferroelectric or nonpolar antiferroelectric packing.

**Figure 1:**
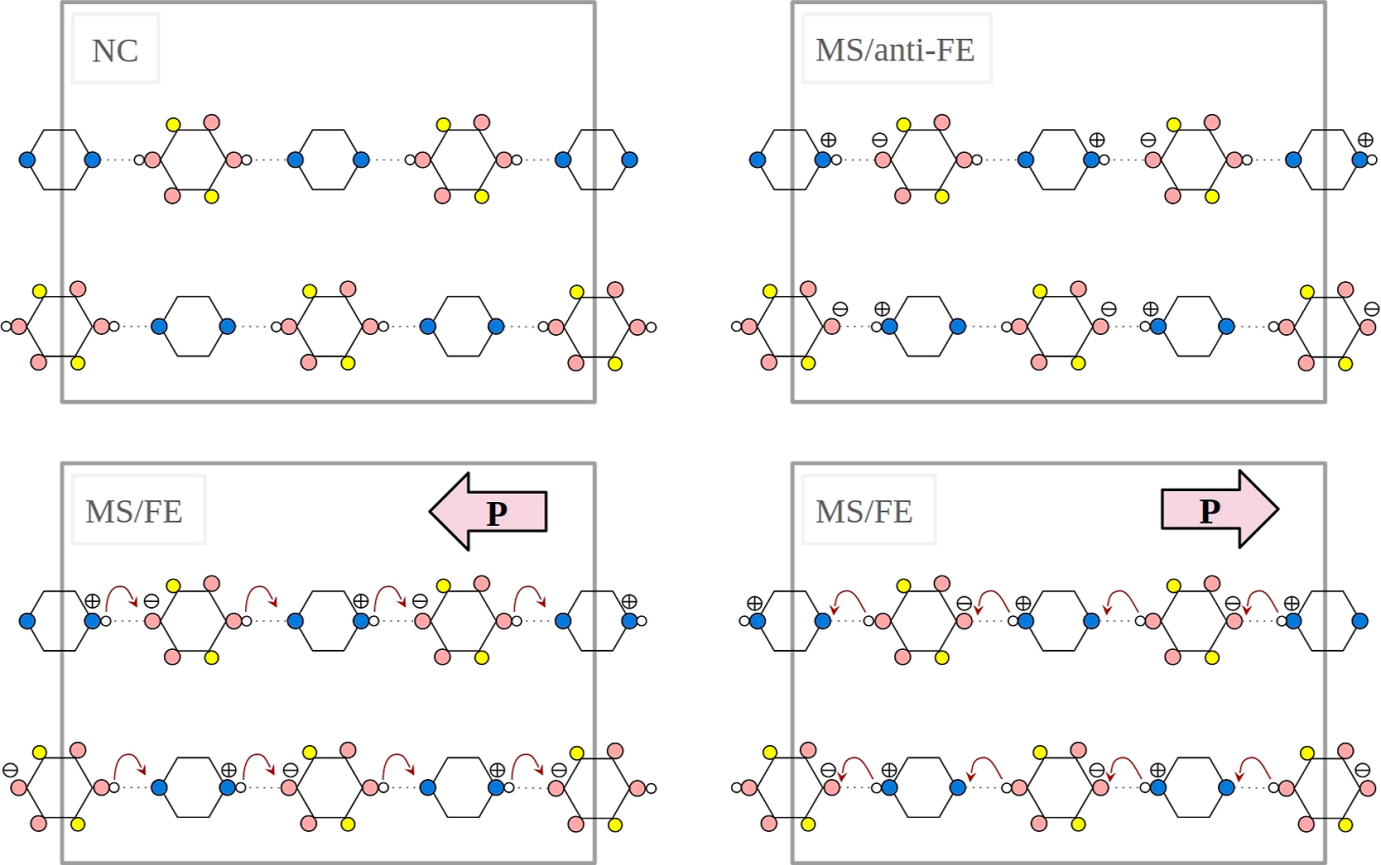
Scenarios for crystal structures with connected proton-transfer paths. The neutral cocrystal (NC) is not ferroelectric, but may exhibit paraelectric properties. The monovalent salt can pack in both antiferroelectric (MS/anti-FE) and ferroelectric (FE) packing.

The difficulty in designing new acid–base proton transfer ferroelectrics can be illustrated by the fact that replacing pyrazine in the bromanilic-pyrazine acid–base crystal^31^ (a proton-transfer ferroelectric) with tetramethyl pyrazine changes both the protonation states and the preferred hydrogen-bonding pattern.^32^ So far, only 10 acid–base PT ferroelectrics are known.^20,24–27,33,34^ These systems all consist of derivatives of haloanilic acid combined with derivatives of phenazine, a tricyclic compound, or bipyridine, i.e., two linked pyridine rings,^24–27^ with PT between oxygen and nitrogen. The largest experimentally reported ***P***_s_ = 5.8 μC/cm^2^ is that of bromanilic acid 2-(3-(pyridin-2-yl)­pyrazin-2-yl)­pyridinium salt,^27^ while the highest computed values fall slightly below 10 μC/cm^2^.^24,28^ These values are far lower than that of the tautomeric compound croconic acid with ***P***_s_ ≈ 30 μC/cm^2^.^14^ The scarcity of PT acid–base ferroelectrics is also illustrated by the fact that none of the 12 PT ferroelectrics uncovered in our recent screening study^28^ of the Cambridge Structural Database (CSD)^35,36^ were acid–base PT salts.

Crystal structure prediction (CSP) methods provide a systematic framework for identifying and assessing the relative stability of the many possible three-dimensional crystal arrangements of molecules or atoms from their basic constituents.^37–40^ In a typical workflow, the configurational space is first explored using efficient sampling methods, followed by local energy minimization to identify relevant metastable structures, which are subsequently ranked according to their formation energies,^41,42^ with the global minimum corresponding to the most stable phase within the chosen energy model. Due to the size of the search space, preliminary CSP landscapes are commonly generated using empirical or semiempirical force fields,^43,44^ followed by refinement of low-energy candidates using higher-level electronic-structure methods, typically density functional theory (DFT) with dispersion corrections.^39,45–50^ Large-scale benchmarks demonstrate that CSP can reliably identify experimentally observed structures for many molecular systems.^51^

In this paper, we used CSP to explore the design of acid–base PT ferroelectric crystals with higher ***P***_s_ than those computed or measured for experimentally known systems. To this end, we considered combinations of single-ring molecular acids and bases. The choice was made due to their potential for higher dipole density than existing systems based on larger molecules. A viable crystal structure requires both stabilization of the MS state (Figure 1) and a crystal packing that supports PT. We explored combinations of molecules capable of supporting PT between oxygen and nitrogen, based on known systems but modified through methyl, fluoro, cyano, and hydroxyl functional groups to influence acidity and crystal packing. The 6 acids and 5 bases considered are shown in Figure 2. Of the 30 combinations, CSP identified three candidate ferroelectric packings and one antiferroelectric. Two of these systems were experimentally investigated and were found to crystallize as NCs rather than as the monovalent salts, as initially predicted, indicating a competition between protonation states.

**Figure 2:**
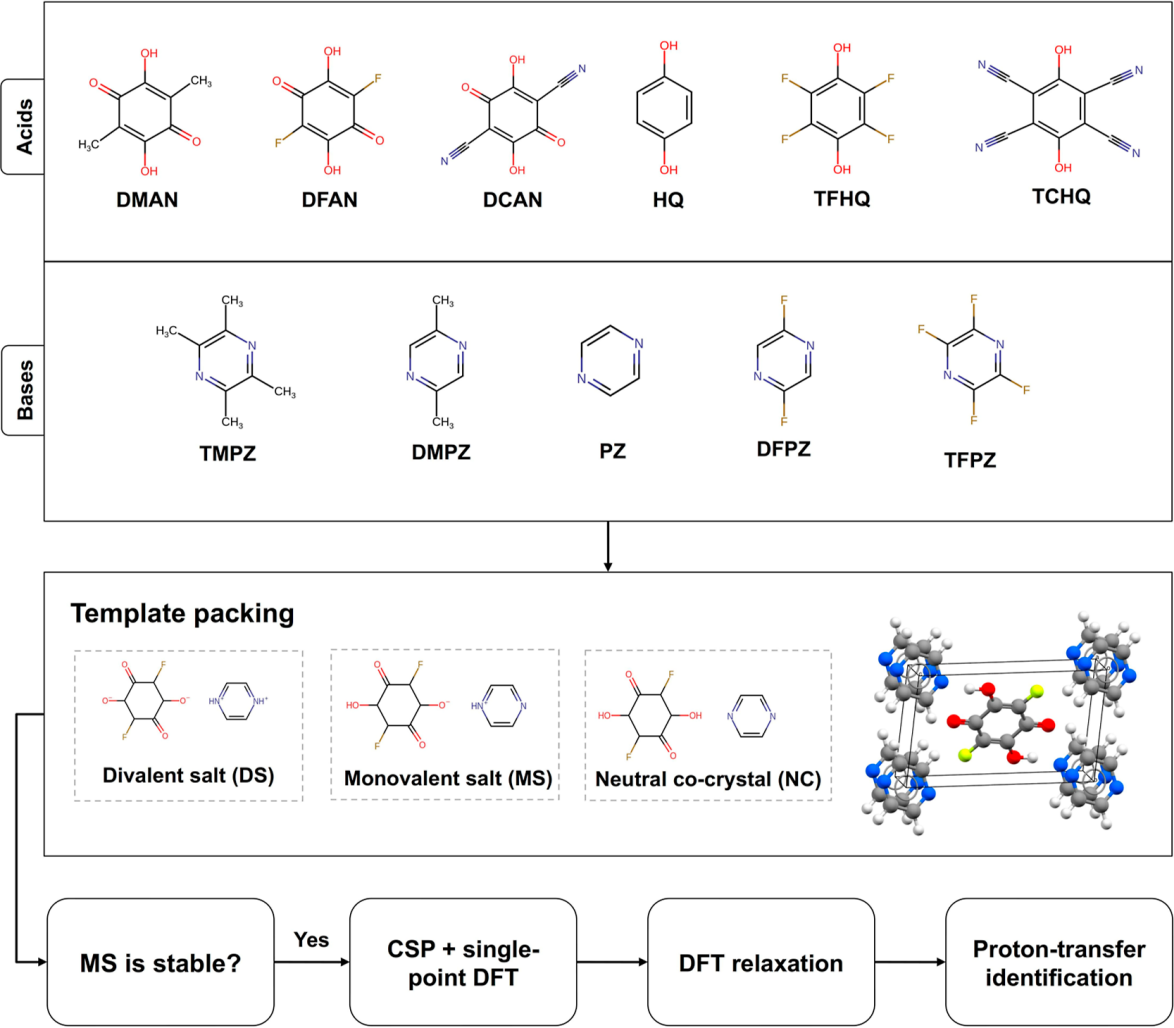
Workflow of the acid–base PT salts design, the acids and bases entering into the computational screening listed (at the top).

## Methods

### Crystal Structure Prediction

The initial CSP stage was performed using rigid molecular geometries optimized with DFT employing the vdW-DF2 functional,^45,52,53^ with a vacuum padding of 15 Å and electrostatic corrections following Neugebauer et al.^54^ The crystal structure configuration space was sampled using the Global Lattice Energy Explorer (glee) protocol,^55^ implemented in molCSPy,^56^ which employs Sobol quasi-random sequences^57^ to sample molecular positions, orientations, and unit cell degrees of freedom. The algorithm adjusts structures with unphysical intermolecular overlaps where feasible, and where not, the structure is discarded.

Lattice energy minimization of the resulting trial structures was carried out using dmacrys,^43^ employing a Buckingham-type atomistic potential with dispersion and repulsion terms from the fit parameter set.^58^ Electrostatic interactions were described using atom-centered multipoles up to rank 4, obtained from distributed multipole analysis (DMA)^59^ of charge densities computed with psi4 ^60^ at the B3LYP/6-311G** level.^61,62^

The crystal structure search was performed over the ten most commonly observed space groups for cocrystals, *P*1, P1̅, *P*2_1_/*c*, *C*2/*c*, *P*2_1_, *P*2_1_2_1_2_1_, *Pbca*, *Pna*2_1_, *C*2, and *Cc*, as well as space groups commonly observed in PT ferroelectric crystals and related antiferroelectrics/paralelectrics: *P*2/*c*, *Pca*2_1_, *Pnn*2, *Pccn*, *Fdd*2, *Iba*2, *P*2, *P*3, *P*6_1_, and *I*4_1_/*a*.^9,10,14,21,24–27^ The quasi-random generation of crystal structures in each space group was terminated upon reaching 20,000 successfully energy-minimized structures or after 100,000 failed generation attempts. Duplicate structures were removed from the final set by comparing simulated powder X-ray diffraction patterns generated with platon,^63^ using constrained dynamic time warping as a similarity metric. Structural similarity to experimentally determined crystal structures was additionally assessed using COMPACK,^64^ quantified by the number of overlapping molecules within a 30% distance and 30° angular tolerance on atom–atom contacts.

### Density Functional Theory Calculations

DFT calculations were performed using the projector augmented-wave (PAW) plane-wave method as implemented in the vasp software package.^65–67^ A plane-wave energy cutoff of 530 eV was used, converging the energy difference per acid–base pair between competing CSP structures to within 5 meV. The Brillouin zone was sampled using a Γ-centered grid with a maximum k-point spacing of (1/25) Å^–1^. Electronic self-consistency was achieved when total energy differences fell below 10^–8^ eV, and ionic relaxations continued until forces fell below 0.01 eV/Å.

The vdW-DF2 functional was used for ranking crystal structures. The choice was made due to its ability to accurately reproduce lattice parameters of organic PT ferroelectrics^28,68^ and molecular crystals more generally.^69–71^ Its accurate PT barrier energies between small organic molecules also motivated the choice,^72^ and we performed a validation study to evaluate its applicability, detailed in the Results section. For the two candidate ferroelectrics that crystallized experimentally as NCs, we additionally examined the sensitivity to the exchange–correlation functional using vdW-DF-cx^73,74^ and the hybrid variant vdW-DF-cx0.^75^ Vibrational free-energy contributions were evaluated within the harmonic approximation using the phonopy package,^76,77^ based on 2 × 2 × 2 supercell configurations and a **q**-sampling of 40 × 40 × 40. The Helmholtz free energy was computed as

1 $$F\left(T\right)=E_{DFT}+F_{vib}\left(T\right)$$

with vibrational contribution given by

2 $$F_{vib}\left(T\right)=\sum_{i}\left[\frac{1}{2}\hbar \omega_{i}+k_{B}T\;\ln\left(1-e^{-\hbar \omega_{i}/k_{B}T}\right)\right]$$

The spontaneous polarization ***P***_s_ was computed for the three candidate ferroelectric structures using the Berry-phase method.^78–80^ The corresponding polarization branch was identified by constructing an adiabatic path from the polar structure to a centrosymmetric reference, as illustrated in Figure 3 and detailed in ref 28 using the molcrys Python tools.^81^

**Figure 3:**
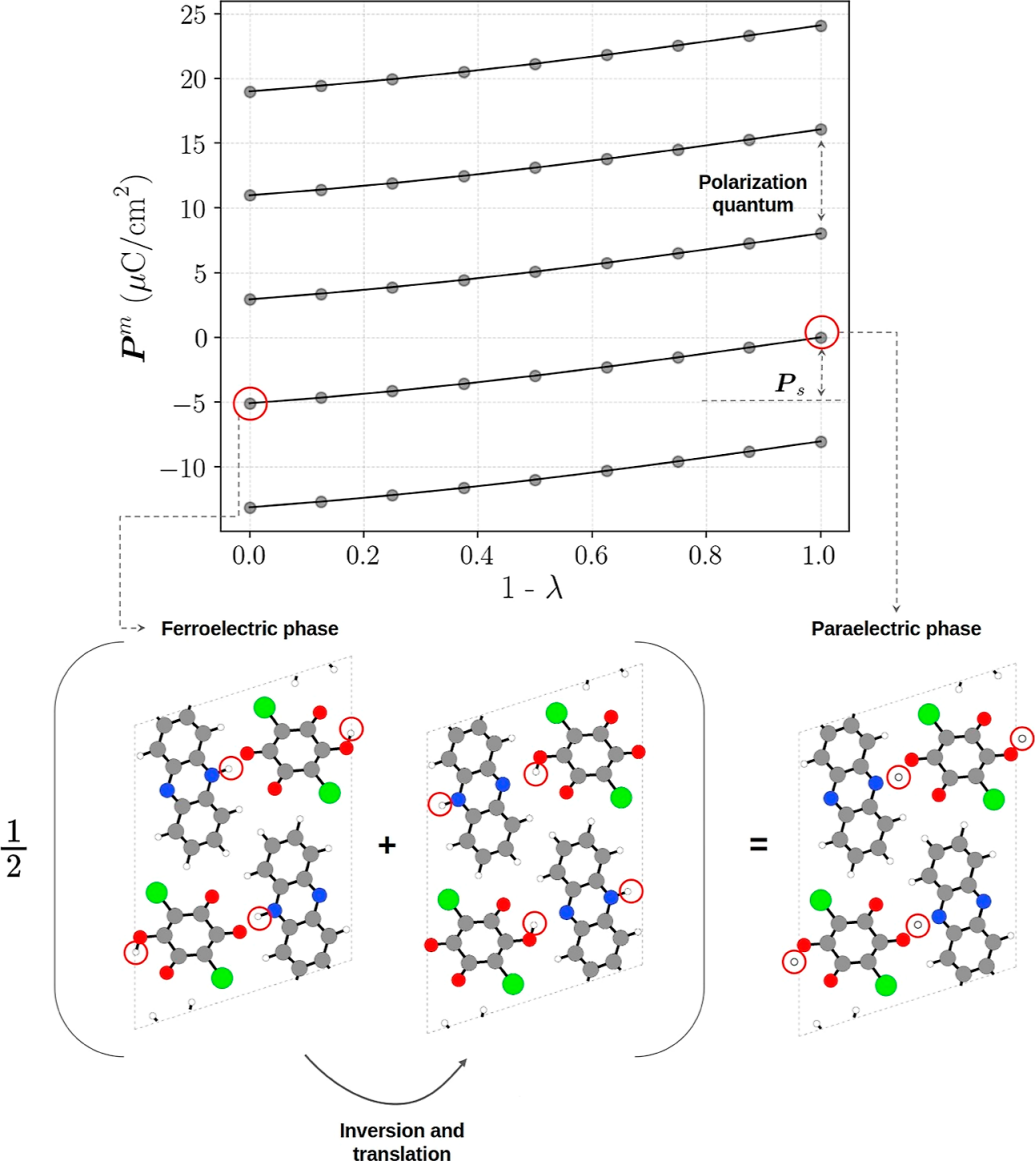
Procedure to identify the polarization branch adiabatically connected to zero in the centrosymmetric limit (illustrated for phenazinium chloranilate). A centrosymmetric reference structure was constructed by inverting the crystal (**r** → −**r**), determining the translation that maximizes overlap with the original structure, and averaging the atomic positions. A continuous interpolation between this reference and the polar structure was then generated, and the polarization was evaluated along this path.

### Crystallization Method

Multiple crystallizations were prepared using the following solvents: ethanol, methanol, acetonitrile, acetone, and water. Difluoroanilic acid was purchased from Ambinter (c/o Greenpharma, France). Pyrazine (PZ) and dimethylpyrazine (DMPZ) were purchased from Sigma-Aldrich. The solvents ethanol, methanol, acetonitrile, and acetone were purchased from Sigma-Aldrich, while water was obtained onsite using a deionizer. Crystallization of difluoroanilic acid (DFAN) and PZ was first attempted by slow evaporation from solutions with a 1:1 molar ratio. A second attempt used slow evaporation from solutions with a 1:2 molar ratio of DFAN to PZ. On the third attempt, crystals were obtained by heated evaporation at 50.0 °C from solutions with a 1:2 molar ratio of DFAN to PZ. For DFAN and DMPZ, crystals were obtained by slow evaporation from solutions with a 1:1 molar ratio at room temperature.

## Results

The Results section follows the CSP design workflow, from validation of the approach, through candidate selection and CSP calculations at different levels of sophistication, to experimental testing and analysis of the theory–experiment discrepancy. The **CSP validations** detail two reference studies, one of a known NC and one of a known PT ferroelectric salt, establishing the reference point for the subsequent design work. The **Cocrystal design** section then defines the design targets and identifies an initial set of acid–base combinations according to their preferred protonation state in template structures. A broad **CSP using DFT energies with fixed geometries** is used to analyze general trends and remove unlikely PT candidates. We then present the **Full CSP** results, divided into **Systems not predicted to be ferroelectric or antiferroelectric**, **PT antiferroelectrics and systems predicted to phase separate**, and **Candidate PT ferroelectric structures**. The **Experimental results** section reports the crystallization and structural characterization of two systems computationally predicted to be PT ferroelectrics. Finally, the **Theory–experiment comparison** analyzes the sensitivity of **salt–cocrystal energetics** to the exchange–correlation functional and vibrational free-energy contributions.

### Crystal Structure Prediction Validation

For assessing the ability to predict acid–base PT salts using CSP, we selected one known hydrogen-bonded NC, 2,3,5,6-tetrafluorobenzene-1,4-diol quinoxaline (CSD refcode: QUWZIS), which adopts a paraelectric configuration in a packing similar to PT ferroelectrics (see Figure 1), and one MS exhibiting PT ferroelectricity, phenazinium chloranilate (CSD refcode: MAMPUM03).^25^ CSP was performed with the molecular components initialized in both NC and MS protonation states. In the initial stage of CSP, the force-field-based dmacrys calculations identified a structure matching the experimental crystal of QUWZIS as the global energy minimum. A match with the experimental structure was also identified for the PT salt (MAMPUM03); however, this structure was ranked 42nd in lattice energy, at approximately 0.07 eV above the global minimum.

Figure 4 summarizes the re-evaluation of lattice energies using DFT. Panels (a) and (b) correspond to the experimental paraelectric NC (QUWZIS), while (c) and (d) correspond to the acid–base ferroelectric (MAMPUM03). In the single-point DFT (panels a and c), the DFT energies were computed without further relaxation using the structures obtained with dmacrys. The energy of zero was set to that of the fully relaxed global minimum (panels b and d).

**Figure 4:**
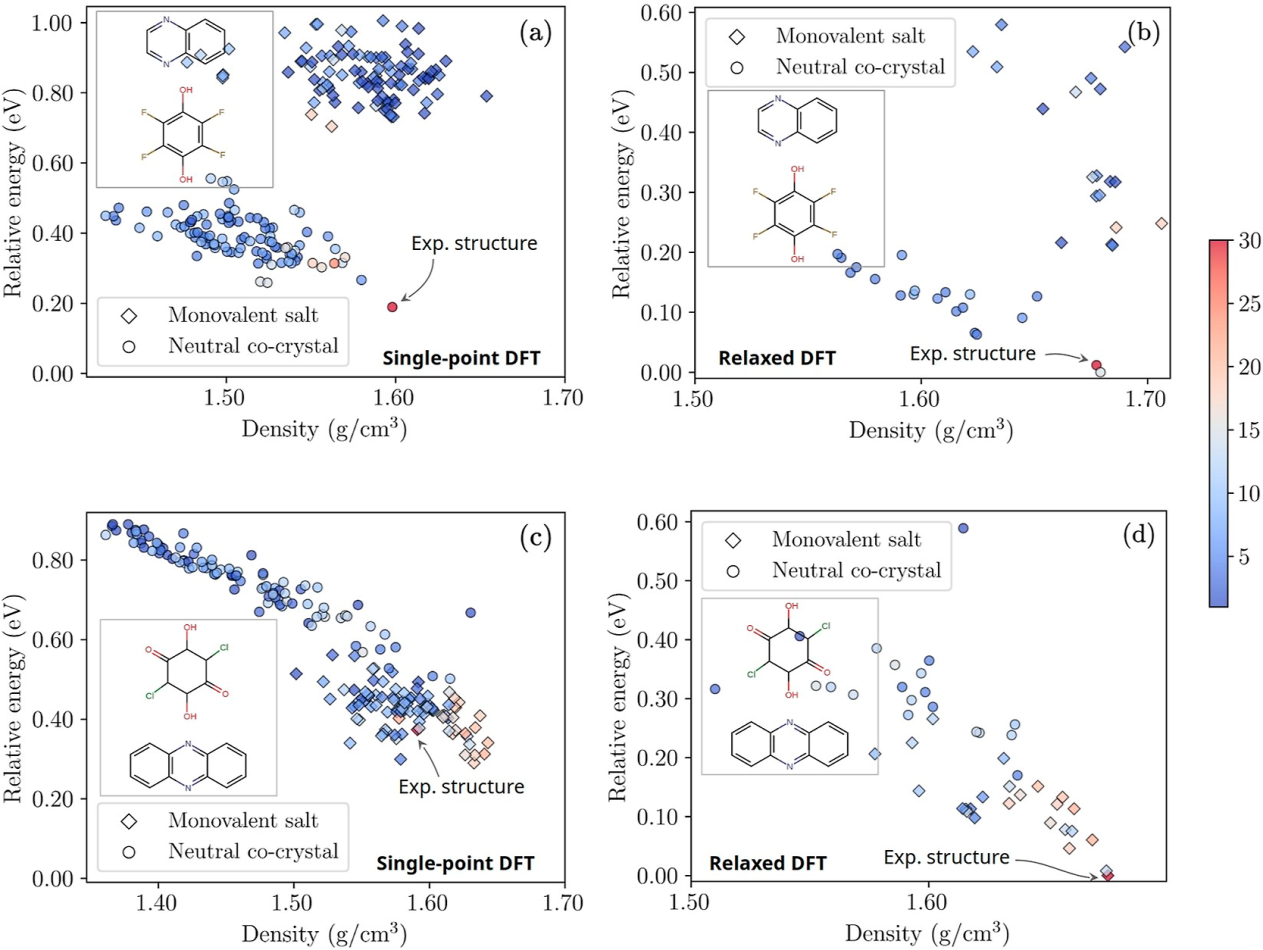
CSP landscapes for two validation systems. Panels (a,b) show energy landscapes after single-point DFT at the force field predicted structure, and after full structure relaxation using DFT, respectively, for 2,3,5,6-tetrafluorobenzene-1,4-diol quinoxaline (experimental structure with CSD reference code QUWZIS). Panels (c,d) are the corresponding landscapes for phenazinium chloranilate (MAMPUM03). The colormap indicates the crystal structure similarity (number of matching molecules) to the experimental crystal structure using compack.

For the former set of structures, good agreement with experiment is found at all theory levels and remains nearly degenerate with the global minimum after DFT relaxation (b). The competing structure also shows high structural similarity to the experimental structure (QUWZIS) and retains the same hydrogen-bonding arrangement. For the latter, the structure ranking successively shows improved agreement with the experiment at the theory level. After full DFT relaxation (d), the computed structure coincides with the global minimum. As in the NC case, the lowest-energy competing structure remains structurally similar to the experimental one. In both cases, relaxed structures show excellent agreement with the experiment. For QUWZIS (panel b), a 30-molecule overlay yields a root-mean-square deviation (RMSD_30_) of 0.126 Å, while for MAMPUM03 (Figure 5), the RMSD_30_ is 0.217 Å.

**Figure 5:**
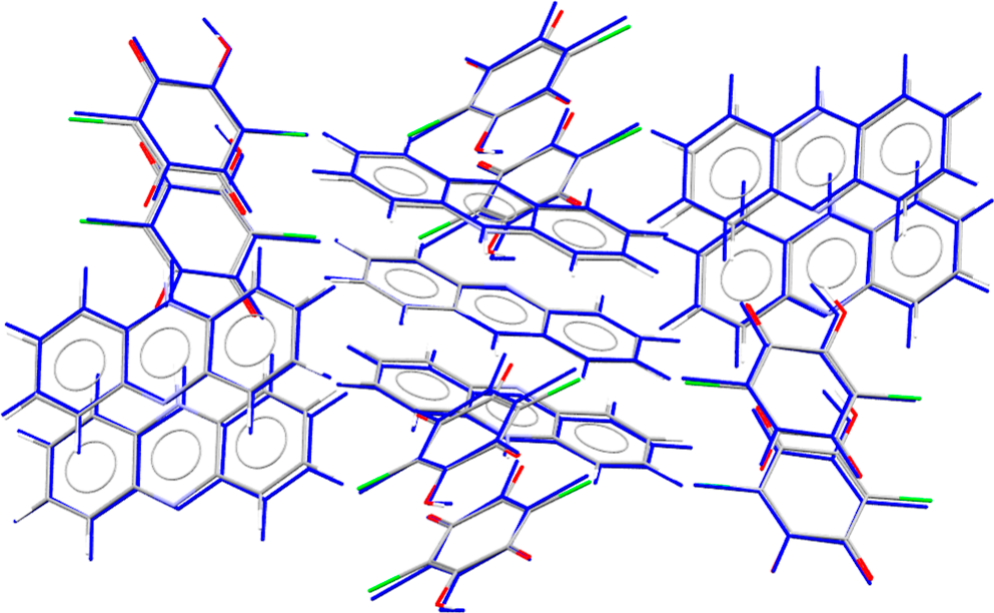
Overlay of the CSP global energy minimum for phenazinium chloranilate (shown in blue) with the experimentally determined crystal structure, CSD reference code MAMPUM03 (shown with atoms colored by element).

### Cocrystal Design

The computational design workflow is outlined in Figure 2. The following acids were considered: **d**i**m**ethyl **an**ilic acid (DMAN), **d**i**f**luoro **an**ilic acid (DFAN), **d**i**c**yano **an**ilic acid (DCAN), **h**ydro**q**uinone (HQ), **t**etra**f**luoro **h**ydro**q**uinone (TFHQ), and **t**etra**c**yano **h**ydro**q**uinone (TCHQ); and the following bases: **t**etra**m**ethyl **p**yra**z**ine (TMPZ), **d**i**m**ethyl **p**yra**z**ine (DMPZ), **p**yra**z**ine (PZ), **d**i**f**luoro **p**yra**z**ine (DFPZ), and **t**etra**f**luoro **p**yra**z**ine (TFPZ). These molecules are symmetric, allowing the formation of pseudosymmetric PT salts.^28^

Structures forming from combinations of acids and bases were assessed according to three criteria for PT ferroelectricity:

1. The crystal must adopt a monovalent salt (MS) configuration and not a neutral cocrystal (NC) or divalent salt (DS) configuration.
2. The hydrogen-bonding pattern should support ferroelectric switching via PT cascades, that is, one-dimensional chains of directional hydrogen bonds must be present.
3. The structure must have a polar space group. Otherwise, with the above being fulfilled, the structure is classified as an antiferroelectric.

To avoid doing CSP for systems unlikely to form MS configurations, we first removed combinations expected to favor NC or DS configurations due to very small or very large acidity differences. While such an assessment can in principle be based on p*K*_*a*_ values, a water environment is not necessarily representative of the crystalline environment. We therefore chose to make this assessment based on protonation states in template crystal structures. For systems with smaller functional groups, such as DFAN, DCAN, PZ, and DFPZ, a tighter template arrangement with the *P*1 space group was adopted from a related compound (pyrazine chloranilic acid) reported in the CSD.^82^ For the remaining systems, a looser packing with the *Pc* space group, obtained from CSP of the largest molecules (TCHQ-TMPZ), was used.

Figure 6 displays the resulting favorability of the protonation states in the template structures. With electron-withdrawing side groups, such as the cyano group in DCAN and TCHQ, and bases with electron-donating groups such as methyl in DMPZ and TMPZ, most of the blue-shaded boxes in the upper panel appear in the lower right part of the matrix. The lower panel shows that molecular combinations involving DCAN tend to donate both protons, i.e., form DS configurations, consistent with the higher acidity of anilic acid rings compared to hydroquinone rings.^83,84^ The 17 systems with dynamically unstable MS configurations were discarded, as were DMAN-PZ and DCAN-TMPZ due to the low energetic favorability of the MS configuration. For DMAN-PZ, the NC is more than 0.3 eV lower in energy per acid–base pair than the MS, while for DCAN-TMPZ, the DS is similarly strongly preferred. More aggressive exclusions at this stage might be warranted, but initial CSP studies revealed that the crystal structure strongly influences the preferred protonation state, as also observed in other studies.^30^

**Figure 6:**
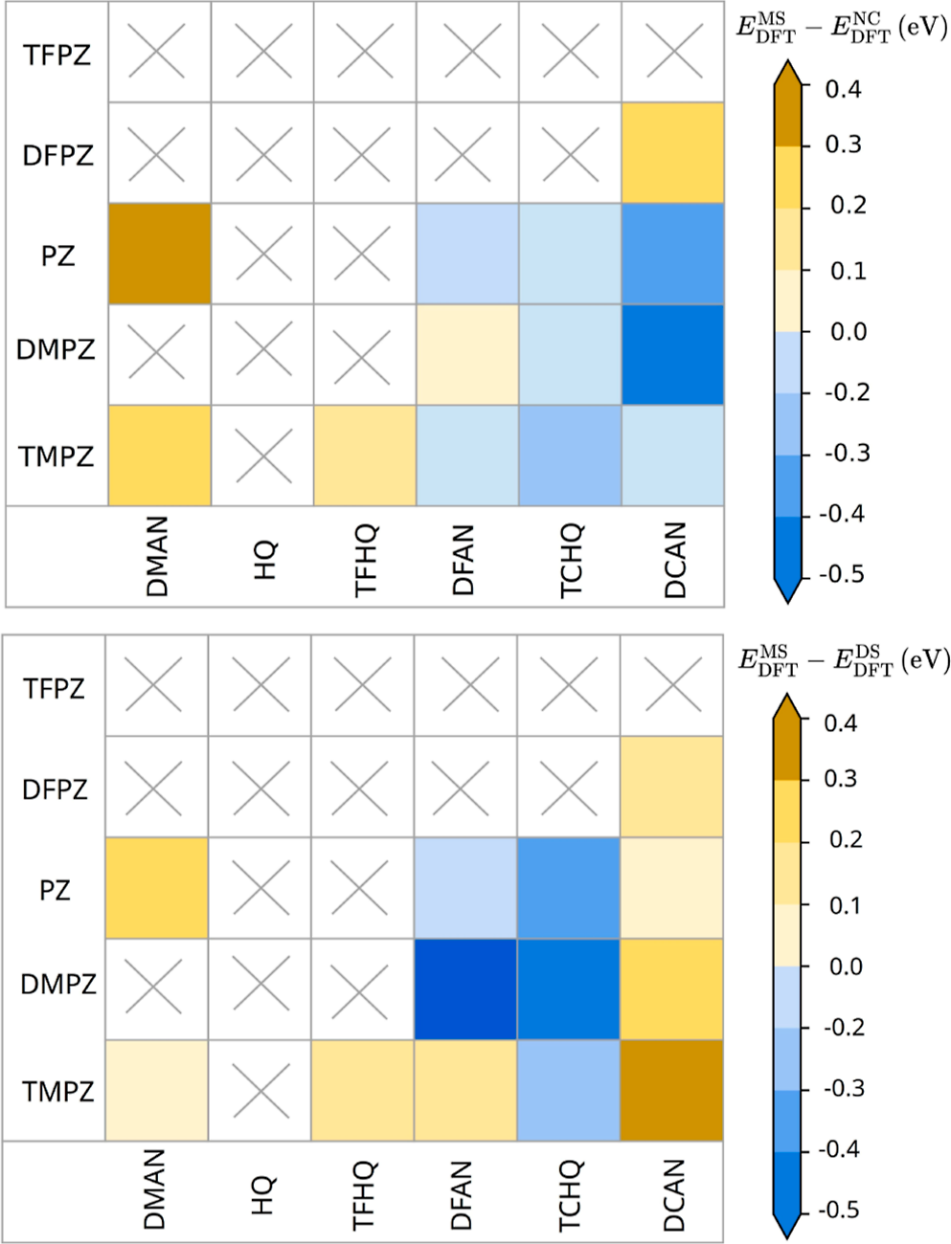
Favorability of the monovalent salt (MS) state relative to the neutral cocrystal (NC) (upper panel) and divalent salt (DS) (lower panel). The color scale indicates the energy difference between MS and NC or DS. The marks X indicate systems where the MS state was found to be dynamically unstable.

To limit the number of computations, CSP was performed only for MS and NC configurations for most systems. However, in cases where a ferroelectric or antiferroelectric packing was identified and the DS configuration was favored over MS in the template analysis, the study was extended to include DS configurations. For predicted ferroelectric or antiferroelectric PT salts, the crystal formation energy was also evaluated by computing reference energies of the corresponding pure acid and base crystals, that is, to obtain formation energies.

### Crystal Structure Prediction Using Density Functional Theory Energies with Fixed Geometries

An initial evaluation of the 11 systems was performed using single-point DFT energies calculated at the DMACRYS force-field geometries of the CSP structures. Figure 7 shows the energy distributions of the 100 lowest-energy MS and 100 lowest-energy NC crystal structures obtained from CSP. Comparison to the template packing results,Figure 6 shows that the trends are generally maintained. However, for DFAN-DMPZ, the MS configuration becomes preferred. The significant overlap in the distributions of NC and MS structures, such as for DMAN-TMPZ, highlights the interplay between crystal structure and preferred protonation state.

**Figure 7:**
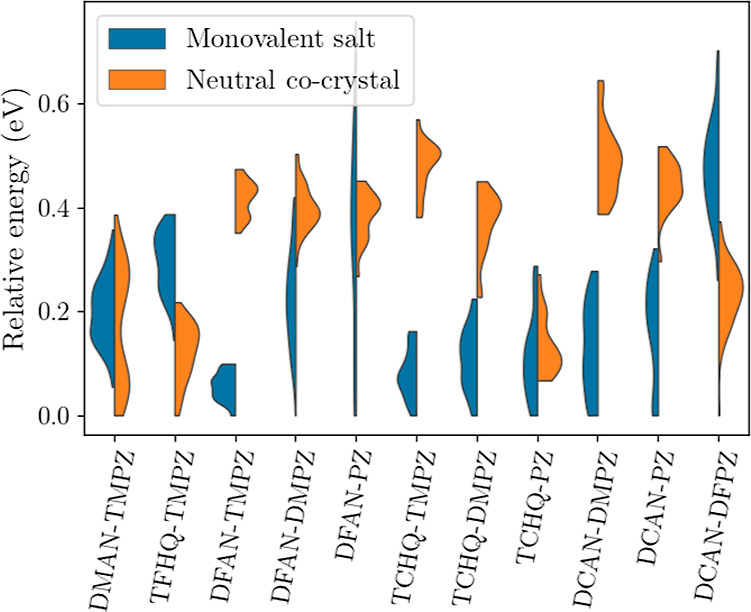
Energy distributions of MS and NC structures obtained from CSP using DFT energies with fixed geometries for the 11 molecular combinations that passed the template screening.

Altogether, MS configurations were preferred for 8 of the 11 systems. For TFHQ-TMPZ and DCAN-DFPZ, most low-energy structures were NCs, with all predicted MS structures at least 0.14 eV higher in energy than the most stable NC structure. These two systems were therefore not reranked by including ionic relaxation.

### Full Crystal Structure Prediction: Systems Not Predicted to be Ferroelectric or Antiferroelectric

CSP with full DFT relaxation was performed for 9 systems. For 4 of these, neither PT ferroelectric nor antiferroelectric crystal structures were preferred. The corresponding CSP landscapes with relaxed energies are shown in Figure 8. For both TCHQ-PZ (a) and TCHQ-DMPZ (b), polar NC crystal structures with connected PT paths were preferred. Being polar, they can in principle be ferroelectric, but they do not support PT switching, as illustrated in Figure 1. Among the higher-energy MS structures, none exhibited connected PT paths. For DCAN-PZ (c), the global minimum and all structures within the typical energetic range of polymorphism^85^ were MS structures without connected PT paths. For DCAN-DMPZ (d), we initially found low-energy MS structures with connected PT paths, with the lowest-energy structure being nonpolar and hence a potential antiferroelectric. However, since the template packing for DCAN-DMPZ clearly favored a DS configuration, we constructed a corresponding DS structure from the lowest-energy MS structure; after DFT relaxation, this DS structure (indicated by the star) was more stable than all MS and NC CSP structures. More stable DS crystal structures may be found with further CSP.

**Figure 8:**
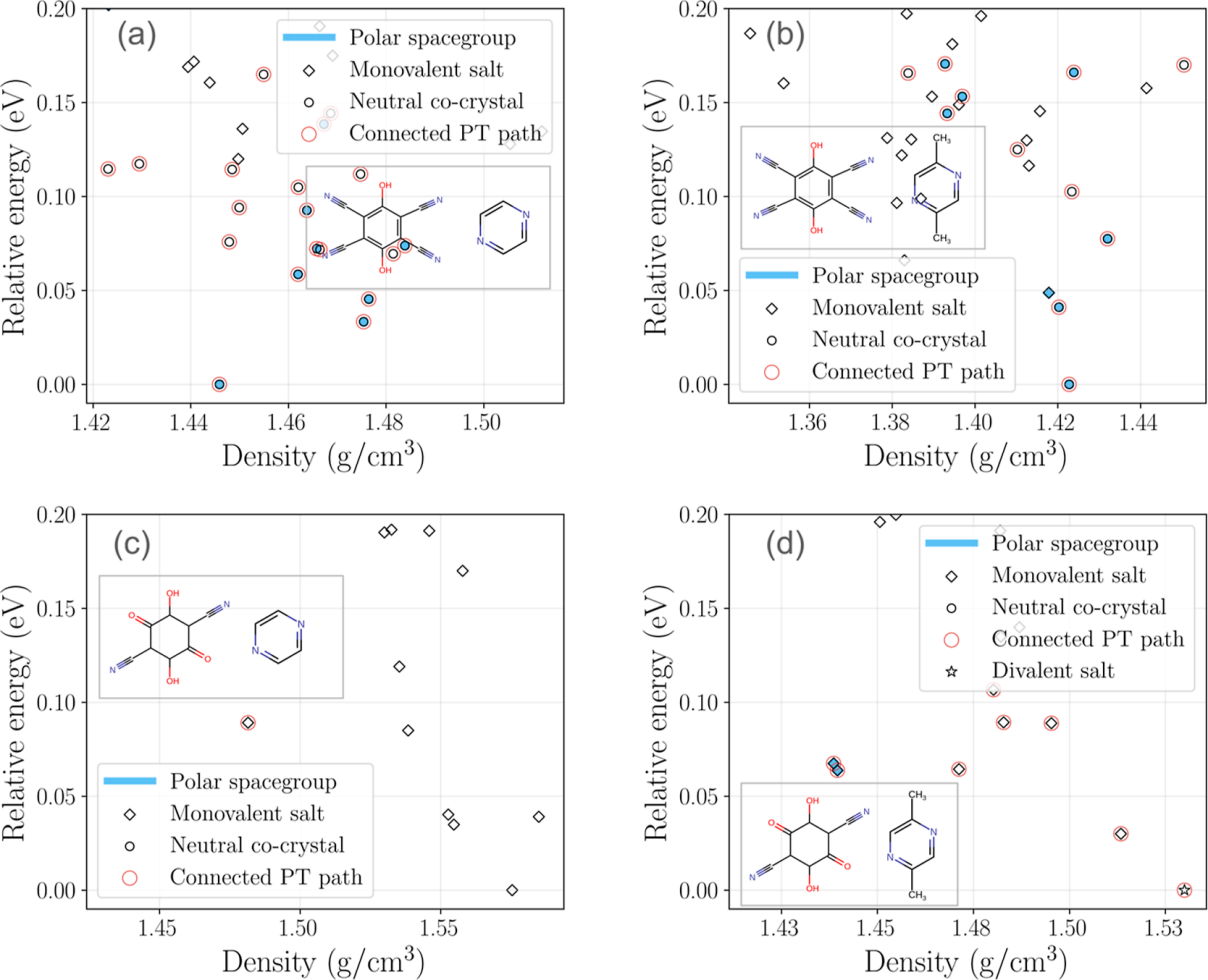
CSP landscapes (DFT relaxed) for the (a) TCHQ-PZ, (b) TCHQ-DMPZ, (c) DCAN-PZ, and (d) DCAN-DMPZ combinations. None of them is ferroelectric.

### Full Crystal Structure Prediction: PT Antiferroelectrics and Phase Separation

Figure 9 shows the CSP landscapes of DMAN-TMPZ (a) and DFAN-TMPZ (b), along with the corresponding predicted global minimum structures. Within the space of cocrystals and salts, both systems have MS global minima with connected PT paths but nonpolar space groups. These conditions support PT antiferroelectricity. However, DMAN-TMPZ has a positive formation energy indicating phase separation. For DFAN-TMPZ, many low-energy structures adopt antiferroelectric crystal packings, including the second-ranked structure with energy very close to the global minimum.

**Figure 9:**
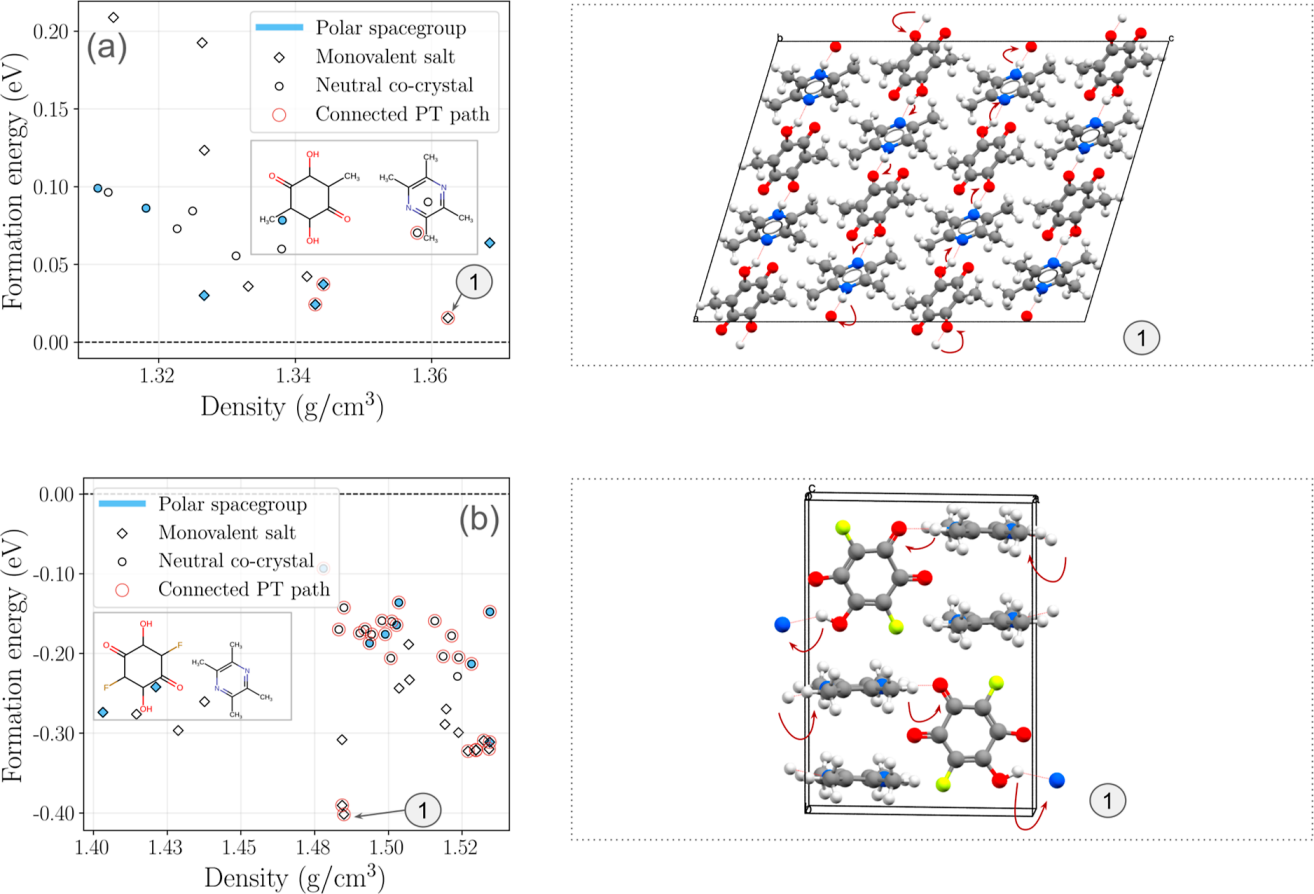
CSP landscapes (DFT relaxed) for (a) DMAN-TMPZ and (b) DFAN-TMPZ. Both have antiferroelectric structures, but DMAN-TMPZ has a positive formation energy and would phase separate.

We also performed CSP studies of the DS configurations for both DMAN-TMPZ and DFAN-TMPZ at the single-point DFT level, since DS configurations were preferred in the template packings. However, their lowest-energy predicted DS structures were 0.7 and 1.7 eV higher than the preferred MS structures for DMAN-TMPZ and DFAN-TMPZ, making DS formation unlikely. These structures were therefore not further relaxed.

### Full Crystal Structure Prediction: Candidate PT Ferroelectric Structures

Figure 10 shows the CSP landscapes and selected associated crystal structures of DFAN-PZ (a), DFAN-DMPZ (b), and TCHQ-TMPZ (c). All three have predicted global energy minima consistent with the conditions for PT ferroelectricity (Figure 1), i.e., MS structures with connected PT paths. For DFAN-PZ (a), most low-energy structures have connected PT paths, but many are also NC, one of which lies only 0.01 eV above the global minimum. For DFAN-DMPZ (b), the structure closest in energy to the global minimum is an antiferroelectric candidate, i.e., an MS with a centrosymmetric space group *P*2_1_/*c* and a connected PT path. All low-energy structures for this system are either potential ferroelectric or antiferroelectric crystals. For TCHQ-TMPZ (c), the low-energy region is occupied mainly by MS structures, but most do not have connected PT paths due to competing synthon introduced by the cyano side groups. Similarly to DFAN-PZ, the energy difference between the global minimum and the second-ranked structure is very small (0.01 eV).

**Figure 10:**
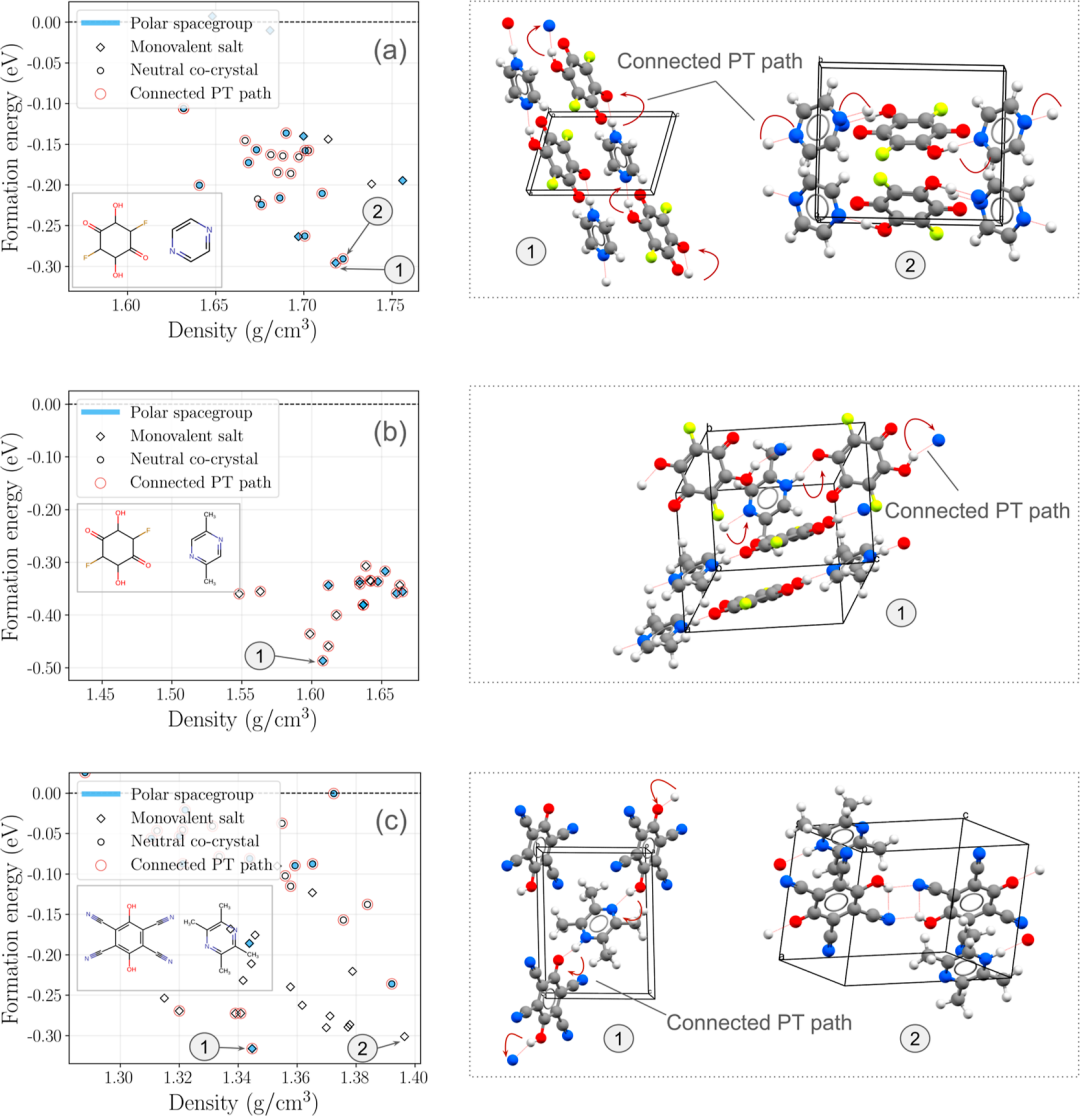
CSP landscapes for (a) DFAN-PZ, (b) DFAN-DMPZ, and (c) TCHQ-TMPZ.

For the predicted ferroelectric structures at the CSP global minimum, we calculated | ***P***_s_| of 14.9, 15.0, and 9.1 μC/cm^2^ for DFAN-PZ, DFAN-DMPZ, and TCHQ-TMPZ, respectively. The two former exceed those reported for existing acid–base systems.

### Experimental Results

Crystallization of DFAN-DMPZ and DFAN-PZ was performed to experimentally test the predictions.

### DFAN and Dimethylpyrazine

Crystals were obtained by slow evaporation at room temperature from solutions of 1:1 molar ratios of DFAN and dimethylpyrazine (DMPZ). Large, parallelogram-shaped plates were obtained by ethanol, methanol, acetonitrile, and acetone solutions. Additionally, much smaller light orange needle and plate-shaped crystals were observed in three of the four successful crystallizations: ethanol, methanol, and acetonitrile. Single-crystal X-ray diffraction (SCXRD) identified the two types of crystals as distinct polymorphs of DFAN-DMPZ cocrystals (NC), and not the desired salt configuration (MS). Both have *Z*′ = 0.5. The most abundant parallelogram-shaped crystals crystallized in the *P*2_1_/*n* monoclinic space group (Form I). The minority light orange needle-shaped crystals, which were much less abundant, crystallized in the P1̅ triclinic space group (Form II). Single crystals of Form I ranged in size from approximately 1 to 400 μm, while those of Form II ranged in size from approximately 50 to 150 μm.

### DFAN and Pyrazine

Crystals were obtained by heated evaporation at 50.0 °C from solutions of 1:2 molar ratios of DFAN and pyrazine (PZ). Two distinct crystal morphologies were observed: Orange needle and plate-shaped crystals were obtained from acetone, ethanol, and methanol solutions and were most abundant. Far less abundant light orange crystals were obtained solely from acetone solutions. As for DFAN-DMPZ, SCXRD identified the two types of crystals as distinct polymorphs of DFAN-PZ NC, not MS, and both have *Z*′ = 0.5. One crystallized in the P1̅ triclinic space group (Form I). The other in the *P*2_1_/c monoclinic space group (Form II). Appendix, Table 2 summarizes the basic crystal data.

**Table 1:** Energy and (Free) Energy Difference MS and NC Configurations for the Respective Global Minima Structures/Networks at 100 K

	Δ*E*_MS–NC_ (meV/f.u.)	
method	DFAN-PZ	DFAN-DMPZ
DF2	-103	-212
DF-cx0	112	-94
DF-cx	-5	-91
DF-cx (free: 100 K)	45	0

**Table 2:** Crystallographic Details for DFAN-DMPZ and DFAN-PZ Crystals

compound	DFAN-DMPZ (I)	DFAN-DMPZ (II)	DFAN-PZ (I)	DFAN-PZ (II)
Formula	C_12_H_10_F_2_N_2_O_4_	C_12_H_10_F_2_N_2_O_4_	C_10_H_6_F_2_N_2_O_4_	C_10_H_6_F_2_N_2_O_4_
*D*_calc*.*_/g cm^–3^	1.593	1.600	1.728	1.712
*m*/mm^–1^	0.140	0.141	0.159	0.157
Formula weight	284.221	284.221	256.167	256.167
Color	orange	orange	orange	orange
Shape	plate	needle	plate	plate
Size/mm^3^	0.06 × 0.03 × 0.02	0.19 × 0.03 × 0.02	0.08 × 0.04 × 0.02	0.06 × 0.05 × 0.02
T/K	100(2)	100(2)	100(2)	100(2)
Crystal system	monoclinic	triclinic	triclinic	monoclinic
Space group	*P*2_1_/*n*	*P*-1	*P*-1	*P*2_1_/*c*
*a*/Å	5.1860(2)	5.1355(1)	5.0461(5)	9.5560(5)
*b*/Å	12.9032(6)	7.9936(1)	6.7442(9)	7.4641(3)
*c*/Å	9.0154(5)	8.2189(2)	8.3671(8)	7.0315(3)
*a*/°	90	67.114(2)	67.138(11)	90
*b*/°	100.801(5)	71.807(2)	80.308(8)	97.761(4)
γ/°	90	85.281(2)	69.914(10)	90
V/Å^3^	592.59(5)	295.001(11)	246.22(5)	496.94(4)
*Z*	2	1	1	2
*Z’*	0.5	0.5	0.5	0.5
Wavelength/Å	0.71073	0.71073	0.71073	0.71073
Radiation type	Mo K_α_	Mo K_*a*_	Mo K_*a*_	Mo K_*a*_
*Q*_min_/°	2.79	2.77	2.64	3.48
*Q*_max_/°	36.31	37.86	30.50	30.99
Measured Refl’s	14313	29471	7043	7844
Indep’t Refl’s	2868	3076	1494	1571
Refl’s I ≥ 2 *s*(I)	2232	2593	1162	1174
*R*_int_	0.0535	0.0365	0.0447	0.0471
Parameters	106	106	91	91
Restraints	0	0	0	0
Largest peak	0.4501	0.4902	0.6053	0.4029
Deepest hole	–0.6125	–0.3120	–0.3992	–0.4522
GooF	1.0299	1.1020	1.0773	1.0122
w*R*_*2*_ (all data)	0.0984	0.0838	0.1254	0.0640
w*R*_*2*_	0.0907	0.0799	0.1155	0.0574
*R*_*1*_ (all data)	0.0563	0.0440	0.0676	0.0597
*R*_*1*_	0.0382	0.0337	0.0496	0.0352

### Theory–Experiment Comparison and Salt–Cocrystal Energetics

The observation of NC state for DFAN-DMPZ was surprising, as the low-energy region of the CSP landscape is dominated by MS structures. However, aside from protonation state, the experimentally observed polymorphs can be directly matched to structures in the CSP landscape. Form I corresponds to the CSP global minimum (rank 0), with an RMSD_30_ of 0.139 Å in atomic positions, indicating essentially identical molecular packing. The second polymorph corresponds to the fourth-lowest-energy CSP structure (rank 3), again with close structural agreement. Both structures are predicted by CSP with good energy ranking, but with the incorrect protonation state.

The disagreement in protonation state for DFAN-PZ is less surprising due to the near degeneracy between NC and MS configurations. Both experimental structures exhibit connected proton-transfer pathways, as do most low-energy CSP structures (Figure 10), consistent with a dominant hydrogen-bonded synthon. Several predicted structures are structurally similar, and the fourth-lowest-energy CSP structure, which is an NC, is in excellent agreement with Form II (RMSD_30_ = 0.152 Å).

The CSP ranking was obtained using vdW-DF2 with fully relaxed atomic positions. However, exchange–correlation-functional sensitivity and vibrational contributions were not accounted for in the rankings. Both effects can alter stability rankings,^86^ and hybrid functionals have been shown to stabilize cocrystals relative to salts and improve the accuracy of predictions.^87–90^

We therefore compared vdW-DF2 with vdW-DF-cx and vdW-DF-cx0. While both vdW-DF2 and vdW-DF-cx yield accurate lattice constants for molecular crystals,^71^ they differ significantly in the exchange enhancement factor *F*_*x*_(*s*),^75,91^ which we have previously identified as critical for proton transfer barrier energetics.^72^

Both vdW-DF-cx and vdW-DF-cx0 substantially reduce the energy difference between the MS and NC configurations (Figure 11, Table 1). vdW-DF-cx0 produces results very similar to vdW-DF-cx for DFAN-DMPS, and therefore we did not explore higher exact exchange fractions. The free energy contributions were computed with vibrational energies obtained with vdW-DF-cx. Including these contributions leads to near degeneracy between MS and NC for DFAN-DMPZ and stabilizes MS for DFAN-PZ.

**Figure 11:**
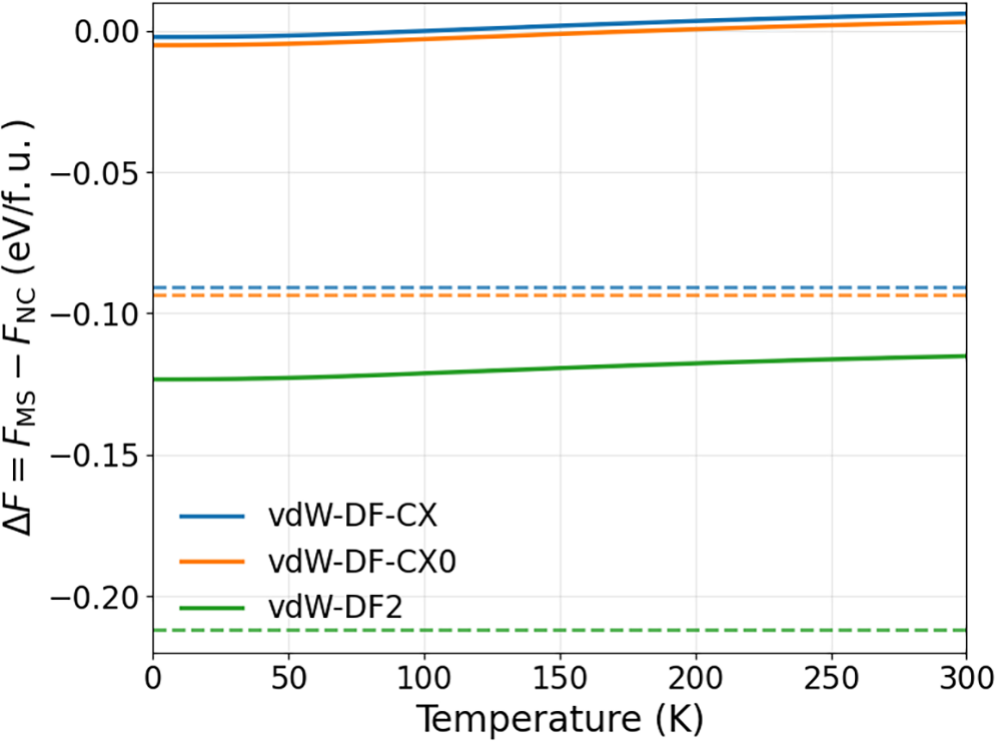
Free-energy difference between MS and NC configurations for the CSP global minimum structure of DFAN-DMPZ (solid curves), including vibrational contributions. Dashed lines show electronic (DFT) energy differences only.

## Conclusions and Outlook

In this study, we used CSP to predict three new PT ferroelectrics, two of which were investigated experimentally; however, NCs rather than the targeted salt configurations were obtained, despite CSP correctly predicting the overall proton-transfer network. The incorrect protonation-state predictions could be traced to exchange–correlation functional sensitivity and vibrational free-energy contributions, both of which strongly affect the MS-NC balance. Thus, the results demonstrate the ability of CSP to predict the observed PT-capable motifs and, thus, to form the basis for computer-guided discovery. However, the findings highlight a fundamental limitation of CSP-based design for functional materials where protonation state is central to functionality, and highlight the need for improved treatments of protonation state energetics, either through more targeted exchange–correlation functionals or approaches incorporating higher-level quantum chemistry or data-driven corrections. It also underscores the importance of including vibrational free-energy contributions when assessing protonation-state preference.

Beyond this disparity, the computational CSP study provides several design insights. Reducing molecular size while preserving proton-transfer functional groups does increase spontaneous polarization, but only 3 out of 30 combinations resulted in predicted ferroelectric candidates. This limited yield reflects the sensitivity of the acidity to molecular modification. Our design strategy attempts to compensate for size-dependent acidity through functionalization, but this introduces competing synthonic and packing arrangements.

In this work, we proposed the use of template crystal structures rather than *pK*_*a*_ to assess protonation-state preference. Template-based predictions reproduce the overall trends seen in CSP, but competing synthons and packing arrangements often lead to different preferred structures. Moreover, the preferred protonation state is strongly coupled to the crystal structure: identical molecular pairs can favor different protonation states depending on the packing. Thus, the prediction of protonation states in molecular crystals is inherently multifaceted, with exchange–correlation functional sensitivity, vibrational free energy, and crystal packing together determining the preferred state.

The fact that only two of the three predicted PT ferroelectric systems could be assessed experimentally in the present study points to a practical limitation in our design strategy. Although the molecular species investigated were chosen from acid and base components represented in the CSD, they were not selected from a list constrained by the ease of purchase or synthesis. A more pragmatic approach to cocrystal design could therefore include purchasability and synthesizability as initial screening criteria so that computational predictions can be better integrated with experimental assessment.

## Data Availability

A dedicated GitLab package gitlab.com/m7582/csp_data describes generated data (with links to different NOMAD uploads), hosts Python scripts used in data analysis and plotting, and describes the workflow for generating CSP landscapes. The complete NOMAD data set is provided at 10.17172/NOMAD/2024.10.22–1.

## Appendix

### Crystallographic Details

Table 2 summarizes the crystallographic parameters obtained from single-crystal X-ray diffraction (SCXRD) for DFAN-DMPZ and DFAN-PZ polymorphs.

## References

[1] Scott J. F. (2007). Applications
of modern ferroelectrics. Science.

[2] Mai M., Ke S., Lin P., Zeng X. (2015). Ferroelectric Polymer
Thin Films
for Organic Electronics. J. Nanomater..

[3] Scott J. F., Paz de Araujo C. A. (1989). Ferroelectric memories. Science.

[4] Martin L. W., Rappe A. M. (2016). Thin-film ferroelectric
materials and their applications. Nat. Rev.
Mater..

[5] Horiuchi, S. ; Ishibashi, S. ; Tokura, Y. In Organic Ferroelectric Materials and Applications; Asadi, K. , Ed.; Woodhead Publishing Series in Electronic and Optical Materials; Woodhead Publishing, 2022; pp 47–84.

[6] Horiuchi S., Ishibashi S. (2020). Hydrogen-Bonded Small-Molecular Crystals Yielding Strong
Ferroelectric and Antiferroelectric Polarizations. J. Phys. Soc. Jpn..

[7] Pan Q., Gu Z.-X., Zhou R.-J., Feng Z.-J., Xiong Y.-A., Sha T.-T., You Y.-M., Xiong R.-G. (2024). The Past 10 Years
of Molecular Ferroelectrics: Structures, Design, and Properties. Chem. Soc. Rev..

[8] Li J., Liu Y., Zhang Y., Cai H.-L., Xiong R.-G. (2013). Molecular Ferroelectrics:
Where Electronics Meet Biology. Phys. Chem.
Chem. Phys..

[9] Horiuchi S., Kagawa F., Hatahara K., Kobayashi K., Kumai R., Murakami Y., Tokura Y. (2012). Above-Room-Temperature
Ferroelectricity and Antiferroelectricity in Benzimidazoles. Nat. Commun..

[10] Horiuchi S., Kobayashi K., Kumai R., Ishibashi S. (2017). Proton tautomerism
for strong polarization switching. Nat. Commun..

[11] Horiuchi S., Tokura Y. (2008). Organic ferroelectrics. Nat.
Mater..

[12] Owczarek M., Hujsak K. A., Ferris D. P., Prokofjevs A., Majerz I., Szklarz P., Zhang H., Sarjeant A. A., Stern C. L., Jakubas R., Hong S., Dravid V. P., Stoddart J. F. (2016). Flexible Ferroelectric Organic Crystals. Nat. Commun..

[13] Song X.-J., Zhang Z.-X., Chen X.-G., Zhang H.-Y., Pan Q., Yao J., You Y.-M., Xiong R.-G. (2020). Bistable State of Protons for Low-Voltage
Memories. J. Am. Chem. Soc..

[14] Horiuchi S., Tokunaga Y., Giovannetti G., Picozzi S., Itoh H., Shimano R., Kumai R., Tokura Y. (2010). Above-Room-Temperature
Ferroelectricity in a Single-Component Molecular Crystal. Nature.

[15] Choi H. S., Li S., Park I.-H., Liew W. H., Zhu Z., Kwon K. C., Wang L., Oh I.-H., Zheng S., Su C., Xu Q.-H., Yao K., Pan F., Loh K. P. (2022). Tailoring
the Coercive Field in Ferroelectric Metal-Free Perovskites by Hydrogen
Bonding. Nat. Commun..

[16] Ai Y., Lv H.-P., Wang Z.-X., Liao W.-Q., Xiong R.-G. (2021). H/F Substitution
for Advanced Molecular Ferroelectrics. Trends
Chem..

[17] Liu H.-Y., Zhang H.-Y., Chen X.-G., Xiong R.-G. (2020). Molecular Design
Principles for Ferroelectrics: Ferroelectrochemistry. J. Am. Chem. Soc..

[18] Krishna G. R., Devarapalli R., Lal G., Reddy C. M. (2016). Mechanically Flexible
Organic Crystals Achieved by Introducing Weak Interactions in Structure:
Supramolecular Shape Synthons. J. Am. Chem.
Soc..

[19] Ding X., Wei C., Wang L., Yang J., Huang W., Chang Y., Ou C., Lin J., Huang W. (2024). Multicomponent Flexible Organic Crystals. SmartMat.

[20] Yamamura Y., Saito E., Saitoh H., Hoshino N., Saito K. (2012). New Organic
Ferroelectrics: Cocrystal of 5,5-Dimethyl-2,2-bipyridine and Bromanilic
Acid. Chem. Lett..

[21] Horiuchi S., Kumai R., Tokura Y. (2011). Hydrogen-Bonding Molecular Chains
for High-Temperature Ferroelectricity. Adv.
Mater..

[22] Horiuchi S., Ishibashi S., Haruki R., Kumai R., Inada S., Aoyagi S. (2020). Metaelectric
multiphase transitions in a highly polarizable
molecular crystal. Chem. Sci..

[23] Horiuchi S., Noda Y., Hasegawa T., Kagawa F., Ishibashi S. (2015). Correlated
Proton Transfer and Ferroelectricity along Alternating Zwitterionic
and Nonzwitterionic Anthranilic Acid Molecules. Chem. Mater..

[24] Horiuchi S., Ishibashi S., Kobayashi K., Kumai R. (2019). Coexistence of normal
and inverse deuterium isotope effects in a phase-transition sequence
of organic ferroelectrics. RSC Adv..

[25] Kumai R., Horiuchi S., Sagayama H., Arima T.-h., Watanabe M., Noda Y., Tokura Y. (2007). Structural Assignment of Polarization
in Hydrogen-Bonded Supramolecular Ferroelectrics. J. Am. Chem. Soc..

[26] Horiuchi S., Kumai R., Tokura Y. (2007). A Supramolecular
Ferroelectric Realized
by Collective Proton Transfer. Angew. Chem.
Int. Ed..

[27] Horiuchi S., Kumai R., Tokura Y. (2013). High-Temperature
and Pressure-Induced
Ferroelectricity in Hydrogen-Bonded Supramolecular Crystals of Anilic
Acids and 2,3-Di­(2-pyridinyl)­pyrazine. J. Am.
Chem. Soc..

[28] Seyedraoufi S., Sødahl E. D., Görbitz C. H., Berland K. (2024). Database mining and
first-principles assessment of organic proton-transfer ferroelectrics. Phys. Rev. Mater..

[29] Sarma B., Saikia B. (2014). Hydrogen Bond Synthon
Competition in the Stabilization
of Theophylline Cocrystals. CrystEngComm.

[30] Childs S. L., Stahly G. P., Park A. (2007). The Salt-Cocrystal Continuum: The
Influence of Crystal Structure on Ionization State. Mol. Pharmaceutics.

[31] Tomura M., Yamashita Y. (2000). One-dimensional
supramolecular tapes in the co-crystals
of 2,5-dibromo-3,6-dihydroxy-1,4-benzoquinone (bromanilic acid) with
heterocyclic compounds containing a pyrazine ring unit. CrystEngComm.

[32] Łuczyńska K., Drużbicki K., Lyczko K., Starosta W. (2014). Complementary optical
and neutron vibrational spectroscopy study of bromanilic acid: 2,3,5,6-tetramethylpyrazine
(1:1) cocrystal. Vib. Spectrosc..

[33] Horiuchi S., Ishii F., Kumai R., Okimoto Y., Tachibana H., Nagaosa N., Tokura Y. (2005). Ferroelectricity near
room temperature
in co-crystals of nonpolar organic molecules. Nat. Mater..

[34] Horiuchi S., Kumai R., Tokunaga Y., Tokura Y. (2008). Proton Dynamics and
Room-Temperature Ferroelectricity in Anilate Salts with a Proton Sponge. J. Am. Chem. Soc..

[35] Groom C. R., Bruno I. J., Lightfoot M. P., Ward S. C. (2016). The Cambridge Structural
Database. Acta. Crystallogr. B.

[36] Allen F. H. (2002). The Cambridge
Structural Database: a quarter of a million crystal structures and
rising. Acta. Crystallogr. B.

[37] Hunnisett L. M., Nyman J., Francia N., Abraham N. S., Adjiman C. S., Aitipamula S., Alkhidir T., Almehairbi M., Anelli A., Anstine D. M. (2024). The Seventh Blind Test
of Crystal Structure Prediction: Structure Generation Methods. Acta Crystallogr. B Struct Sci. Cryst. Eng. Mater..

[38] Hunnisett L. M., Francia N., Nyman J., Abraham N. S., Aitipamula S., Alkhidir T., Almehairbi M., Anelli A., Anstine D. M., Anthony J. E. (2024). The
Seventh Blind Test of Crystal Structure
Prediction: Structure Ranking Methods. Acta
Crystallogr. B Struct Sci. Cryst. Eng. Mater..

[39] Bowskill D. H., Sugden I. J., Konstantinopoulos S., Adjiman C. S., Pantelides C. C. (2021). Crystal
Structure Prediction Methods for Organic Molecules: State of the Art. Annu. Rev. Chem. Biomol. Eng..

[40] Beran G. J. O. (2023). Frontiers
of Molecular Crystal Structure Prediction for Pharmaceuticals and
Functional Organic Materials. Chem. Sci..

[41] Price S. L. (2018). Is zeroth
order crystal structure prediction (CSP0) coming to maturity? What
should we aim for in an ideal crystal structure prediction code?. Faraday Discuss..

[42] Woodley S. M., Day G. M., Catlow R. (2020). Structure
prediction of crystals,
surfaces and nanoparticles. Philos. Trans. R.
Soc. A.

[43] Price S. L., Leslie M., Welch G. W. A., Habgood M., Price L. S., Karamertzanis P. G., Day G. M. (2010). Modelling organic
crystal structures
using distributed multipole and polarizability-based model intermolecular
potentials. Phys. Chem. Chem. Phys..

[44] Price S. L. (2014). Predicting
Crystal Structures of Organic Compounds. Chem.
Soc. Rev..

[45] Berland K., Cooper V. R., Lee K., Schröder E., Thonhauser T., Hyldgaard P., Lundqvist B. I. (2015). Van Der
Waals Forces in Density Functional Theory: A Review of the vdW-DF
Method. Rep. Prog. Phys..

[46] Thonhauser T., Zuluaga S., Arter C. A., Berland K., Schröder E., Hyldgaard P. (2015). Spin Signature of Nonlocal Correlation Binding in Metal-Organic
Frameworks. Phys. Rev. Lett..

[47] Tkatchenko A., Scheffler M. (2009). Accurate Molecular Van Der Waals Interactions from
Ground-State Electron Density and Free-Atom Reference Data. Phys. Rev. Lett..

[48] Grimme S., Antony J., Ehrlich S., Krieg H. (2010). A Consistent and Accurate *Ab Initio* Parametrization
of Density Functional Dispersion
Correction (DFT-D) for the 94 Elements H-Pu. J. Chem. Phys..

[49] Otero-de-la-Roza A., Johnson E. R. (2012). A Benchmark for
Non-Covalent Interactions in Solids. J. Chem.
Phys..

[50] Day, G. M. Supramolecular Chemistry; John Wiley & Sons, Ltd, 2012.

[51] Taylor C. R., Butler P. W. V., Day G. M. (2025). Predictive
Crystallography at Scale:
Mapping, Validating, and Learning from 1000 Crystal Energy Landscapes. Faraday Discuss..

[52] Lee K., Murray E. D., Kong L., Lundqvist B. I., Langreth D. C. (2010). Higher-accuracy van der Waals density
functional. Phys. Rev. B.

[53] Berland K., Cooper V. R., Lee K., Schröder E., Thonhauser T., Hyldgaard P., Lundqvist B. I. (2015). Van der
Waals forces in density functional theory: a review of the vdW-DF
method. Rep. Prog. Phys..

[54] Neugebauer J., Scheffler M. (1992). Adsorbate-Substrate and Adsorbate-Adsorbate Interactions
of Na and K Adlayers on Al(111). Phys. Rev.
B.

[55] Case D. H., Campbell J. E., Bygrave P. J., Day G. M. (2016). Convergence Properties
of Crystal Structure Prediction by Quasi-Random Sampling. J. Chem. Theory Comput..

[56] mol-CSPy GitLab. (Accessed March 18, 2025), https://gitlab.com/mol-cspy/mol-cspy.

[57] Sobol’ I. (1967). On the distribution
of points in a cube and the approximate evaluation of integrals. USSR Comput. Math. & Math. Phys..

[58] Coombes D. S., Price S. L., Willock D. J., Leslie M. (1996). Role of Electrostatic
Interactions in Determining the Crystal Structures of Polar Organic
Molecules. A Distributed Multipole Study. J.
Phys. Chem..

[59] Stone A., Alderton M. (1985). Distributed multipole
analysis. Mol. Phys..

[60] Smith D. G. A., Burns L. A., Simmonett A. C., Parrish R. M., Schieber M. C., Galvelis R., Kraus P., Kruse H., Di Remigio R., Alenaizan A. (2020). P si4 1.4: Open-source Software for High-Throughput
Quantum Chemistry. J. Chem. Phys..

[61] Becke A. D. (1993). Density-functional
thermochemistry. III. The role of exact exchange. J. Chem. Phys..

[62] Krishnan R., Binkley J. S., Seeger R., Pople J. A. (1980). Self-consistent
molecular orbital methods. XX. A basis set for correlated wave functions. J. Chem. Phys..

[63] Spek A. L. (2003). Single-crystal
structure validation with the program *PLATON*. J. Appl. Crystallogr..

[64] Chisholm J. A., Motherwell S. (2005). COMPACK: a
program for identifying crystal structure
similarity using distances. J. Appl. Crystallogr..

[65] Kresse G., Hafner J. (1993). Ab initio molecular
dynamics for liquid metals. Phys. Rev. B.

[66] Kresse G., Furthmüller J. (1996). Efficiency of ab-initio total energy
calculations for
metals and semiconductors using a plane-wave basis set. Comput. Mater. Sci..

[67] Kresse G., Furthmüller J. (1996). Efficient
iterative schemes for ab initio total-energy
calculations using a plane-wave basis set. Phys.
Rev. B.

[68] Lee K., Kolb B., Thonhauser T., Vanderbilt D., Langreth D. C. (2012). Structure and energetics of a ferroelectric
organic
crystal of phenazine and chloranilic acid. Phys.
Rev. B.

[69] Chakraborty D., Berland K., Thonhauser T. (2020). Next-Generation
Nonlocal van Der
Waals Density Functional. J. Chem. Theory Comput..

[70] Sødahl E. D., Walker J., Berland K. (2023). Piezoelectric Response
of Plastic
Ionic Molecular Crystals: Role of Molecular Rotation. Cryst. Growth Des..

[71] Jenkins T., Berland K., Thonhauser T. (2025). Van Der Waals
Density Functional
for Molecular Crystals. Phys. Rev. B.

[72] Seyedraoufi S., Berland K. (2022). Improved proton-transfer barriers with van der Waals
density functionals: Role of repulsive non-local correlation. J. Chem. Phys..

[73] Berland K., Hyldgaard P. (2014). Exchange Functional
That Tests the Robustness of the
Plasmon Description of the van Der Waals Density Functional. Phys. Rev. B.

[74] Berland K., Arter C. A., Cooper V. R., Lee K., Lundqvist B. I., Schröder E., Thonhauser T., Hyldgaard P. (2014). van der Waals
density functionals built upon the electron-gas tradition: Facing
the challenge of competing interactions. J.
Chem. Phys..

[75] Berland K., Jiao Y., Lee J.-H., Rangel T., Neaton J. B., Hyldgaard P. (2017). Assessment of Two Hybrid van Der Waals Density Functionals
for Covalent and Non-Covalent Binding of Molecules. J. Chem. Phys..

[76] Togo A. (2023). First-Principles
Phonon Calculations with Phonopy and Phono3py. J. Phys. Soc. Jpn..

[77] Togo A., Chaput L., Tadano T., Tanaka I. (2023). Implementation Strategies
in Phonopy and Phono3py. J. Phys.: Condens.
Matter.

[78] King-Smith R. D., Vanderbilt D. (1993). Theory of Polarization of Crystalline
Solids. Phys. Rev. B.

[79] Resta R. (1992). Theory of
the Electric Polarization in Crystals. Ferroelectr.

[80] Resta R. (1994). Macroscopic
Polarization in Crystalline Dielectrics: the Geometric Phase Approach. Rev. Mod. Phys..

[81] Berland, K. ; Sødahl, E. D. ; Seyedraoufi, S. . Molcrys 2023. https://gitlab.com/m7582/molcrys/ (accessed 2026-07-09).

[82] Liu H., Gutmann M. J., Stokes H. T., Campbell B. J., Evans I. R., Evans J. S. O. (2019). Supercolossal
Uniaxial Negative Thermal Expansion in
Chloranilic Acid Pyrazine, CA-Pyz. Chem. Mater..

[83] Beale R. N., Liberman A. (1950). The determination of
the dissociation constants of
very sparingly soluble weak electrolytes. J.
Chem. Soc..

[84] Mostafa S. I. (1999). Complexes
of 2,5-dihydroxy-1,4-benzoquinone and chloranilic acid with second
and third row transition elements. Transit.
Met. Chem..

[85] Nyman J., Day G. M. (2015). Static and Lattice
Vibrational Energy Differences between
Polymorphs. CrystEngComm.

[86] Hoja J., Tkatchenko A. (2018). First-Principles Stability Ranking of Molecular Crystal
Polymorphs with the DFT+MBD Approach. Faraday
Discuss..

[87] LeBlanc L. M., Dale S. G., Taylor C. R., Becke A. D., Day G. M., Johnson E. R. (2018). Pervasive Delocalisation
Error Causes Spurious Proton
Transfer in Organic Acid–Base Co-Crystals. Angew. Chem..

[88] Bal K. M., Collas A. (2024). Charting the Salt–Cocrystal
Continuum of Acid–Base
Multicomponent Crystals with Hybrid Density Functional Theory. CrystEngComm.

[89] Beran G. J. O. (2025). From
Polymorphs to Cocrystals and Salts: Successfully Predicting Axitinib’s
Challenging Crystal Forms. Chem. Sci..

[90] Perry C. J., Ramos S. A., Phelps M. C., Mueller L. J., Beran G. J. O. (2025). Taming
Tautomerism in Organic Crystal Structure Prediction. J. Am. Chem. Soc..

[91] Jenkins T., Berland K., Thonhauser T. (2021). Reduced-Gradient Analysis of van
Der Waals Complexes. Electron. Struct..

